# Medical Journals

**Published:** 1859-09

**Authors:** 


MEDICAL JOURNALS.—We have received the second number of the *Cleveland Medical Gazette*, a new monthly periodical, edited by Dr. G. C. E. Weber, Professor of Surgery in the Cleveland Medical College. We place it on our exchange list with pleasure, and would thank the editor for the first number.

Dr. T. S. Sullivan has retired from the editorship of the *Savannah Journal of Medicine* which will in future be conducted by Dr. Juriah Harriss and Dr. R. D. Arnold.

Dr. Currey having withdrawn from the *Nashville Monthly Record*, it will hereafter be edited by Professor Wright, assisted by Professors Callender and Maddin.

*The Dental News Letter*, an able quarterly, will from the first of August be issued as a monthly, under its new name of "*The Dental Cosmos*." Dr. McCurdy dissolves his connection with it, and its editorial management will be supervised by Dr. J. D. White, Dr. J. H. McQuillen, and Dr. G. J. Ziegler. From the well-known qualifications of the editors, we predict for it much success.

We have received from Messrs. Sutherland and Knox the fourth number of the *Edinburgh Veterinary Review and Annals of Comparative Physiology*. From the names of its contributors, and the style and value of its articles, we cannot for an instant doubt that it will be warmly received by all those interested in this department. The publishers will oblige us by sending the back numbers.

